# ABA Biosynthesis- and Signaling-Related Gene Expression Differences between Sweet Cherry Fruits Suggest Attenuation of ABA Pathway in Bicolored Cultivars

**DOI:** 10.3390/plants12132493

**Published:** 2023-06-29

**Authors:** Orlando Acevedo, Claudio Ponce, Macarena Arellano, Salvatore Multari, Esther Carrera, José Manuel Donoso, Stefan Martens, Nathalie Kuhn, Lee A. Meisel

**Affiliations:** 1 Instituto de Nutrición y Tecnología de los Alimentos, Universidad de Chile, Macul 7830490, Chile; 2 Facultad de Ciencias Agronómicas y de los Alimentos, Pontificia Universidad Católica de Valparaíso, Valparaíso 2340025, Chile; 3 Department of Food Quality and Nutrition, Fondazione Edmund Mach, Via E. Mach 1, 38010 San Michele all’Adige, Trentino, Italy; 4 Instituto de Biología Molecular y Celular de Plantas (IBMCP), CSIC-Universidad Politécnica de Valencia, 46022 Valencia, Spain; 5 Instituto de Investigaciones Agropecuarias, Centro Regional INIA Rayentué, Rengo 2940000, Chile

**Keywords:** abscisic acid, anthocyanins, bicolored cherries, fruit coloring, IAD, non-climacteric, polyphenolics, ripening, *Prunus avium*

## Abstract

Fruit development involves exocarp color evolution. However, signals that control this process are still elusive. Differences between dark-red and bicolored sweet cherry cultivars rely on MYB factor gene mutations. Color evolution in bicolored fruits only occurs on the face receiving sunlight, suggesting the perception or response to color-inducing signals is affected. These color differences may be related to synthesis, perception or response to abscisic acid (ABA), a phytohormone responsible for non-climacteric fruit coloring. This work aimed to determine the involvement of ABA in the coloring process of color-contrasting varieties. Several phenolic accumulation patterns differed between bicolored ‘Royal Rainier’ and dark-red ‘Lapins’. Transcript abundance of ABA biosynthetic genes (*PavPSY*, *PavZEP* and *PavNCED1*) decreased dramatically from the Pink to Red stage in ‘Royal Rainier’ but increased in ‘Lapins’, which correlated with a higher ABA content in this dark-red cultivar. Transcripts coding for ABA signaling (*PavPP2Cs*, *PavSnRKs* and *PavMYB44.1*) were almost undetectable at the Red stage in ‘Royal Rainier’. Field trials revealed that ‘Royal Rainier’ color development was insensitive to exogenous ABA, whereas it increased in ‘Lapins’. Furthermore, ABA treatment only increased transcript levels of signaling genes in ‘Lapins’. Further studies may address if the ABA pathway is attenuated in bicolor cultivars.

## 1. Introduction

Anthocyanins are the compounds that contribute to fruit color evolution during fruit development and ripening [1,2,3,4,5,6]. They are synthesized in the exocarp cell layer from precursors produced in the phenylpropanoid pathway [7,8]. More expressed structural genes, e.g., *PAL*, *CHI*, *DFR*, *LDOX*, and *UFGT*, are associated with the phenotypic differences between dark-colored (red or blue) and non-colored (white or yellow) cultivars [9,10,11].

Regulation of the structural genes of this pathway occurs by the interaction of DNA-binding R2R3-MYB transcription factors and MYC-like basic helix–loop–helix (bHLH) and WD40-repeat proteins [12]. There is evidence in several fruit models that MYBs are essential in fruit color evolution [13]. For instance, in apple (*Malus domestica*), the R2R3-MYB *MdMYB10* alleles were expressed at higher levels in the red cultivars than in green ones [14,15]. In grape (*Vitis vinifera*), a retrotransposon-induced mutation in the promoter of the *VvMYBA1* gene is associated with a lower anthocyanin content in white cultivars [16].

The regulation of anthocyanin production relies on several external factors, such as light and temperature [4]. However, internal signals, including phytohormones such as abscisic acid (ABA), are also relevant, which is key for ripening initiation in non-climacteric species [17,18,19]. ABA controls the expression of genes involved in fruit color evolution. Kadomura-Ishikawa et al. (2015) [20] have shown that the expression of *FaMYB10,* an important color evolution gene, was independently regulated by light and ABA. In sweet cherry (*Prunus avium*), the treatment with an inhibitor of ABA biosynthesis reduced the expression of several anthocyanin structural genes and the anthocyanin content [18]. 

In the white clover, the uptake of secondary metabolites, Benzoxazinoids, substantially increases the accumulation of flavonoids and ABA [6]. In strawberry, silencing *FaNCED1*—a gene encoding a key enzyme for ABA biosynthesis—caused less expression of *FaMYB10* and reduced anthocyanin content [20]. This evidence shows that ABA plays an important modulatory role in fruit color evolution in fruits and the accumulation of flavonoids in other plant organisms.

Ethylene plays a minor role in fruit ripening-related processes, such as coloration, in the non-climacteric sweet cherry. Anthocyanin accumulation coincides with a dramatic increase in endogenous ABA levels [21,22,23]. Exogenous ABA treatment *ex planta* and *in planta* increase fruit anthocyanin content [13,19,23]. The sweet cherry color evolution during ripening involves de-greening and the accumulation of carotenoids, producing a straw-yellow color. Subsequently, the color of the fruit transitions to a Pink stage due to the anthocyanin production. Some cultivars can accumulate high levels of anthocyanins, evolving into a red-, mahogany- or black-colored fruit at maturity [18,21]; other cultivars remain pink, bicolored and even yellow [10]. 

Analyses of the molecular differences between bicolored and dark-red sweet cherry cultivars are scarce. Dark-red cultivars have a higher abundance of anthocyanin structural gene transcripts when compared to bicolored cultivars [9,10]. MYB transcription factors may also be relevant, as they are anthocyanin-promoting genes in Rosaceous crops [24]. PavMYBA interacts with the bHLH transcription factors to activate anthocyanin structural gene promoters in sweet cherry [18]. *PavMYB10* expresses at higher levels in dark fruits when compared to pink-bicolored fruits [24,25]. Shen et al. (2014) found that silencing of *PavMYBA*, a variant of the *PavMYB10* gene, yielded less anthocyanin accumulation. Additionally, bicolor cultivars are homozygous for a deletion in the third exon of *PavMYB10*, whereas dark-red cultivars are heterozygous for this deletion [10]. Despite these previous studies, a functional demonstration that this deletion is the only cause of color differences between these cultivars is lacking. 

This work aimed to determine to what extent the ABA pathway is involved in color differences between dark-red and bicolored cultivars. Evidence shows that ABA controls the expression of several anthocyanin structural genes and *PavMYB10* in sweet cherry [18]. The anthocyanin regulatory network in response to light in bicolored cherries included ABA signaling genes [26]. However, differences in ABA-related genes between dark and light cultivars and their response to exogenous ABA have not been assessed. Therefore, we used a directed metabolic profile to quantify phenolic compound accumulation in a dark-red and a bicolored cultivar, quantified endogenous ABA levels and analyzed the expression of genes involved in ABA biosynthesis and signaling. Furthermore, we assessed the effect of ABA treatment at the physiological and molecular levels in these two contrasting cultivars. This work provides new evidence for dissecting the control of color evolution during ripening in sweet cherry fruits.

## 2. Results

**Figure 1:**
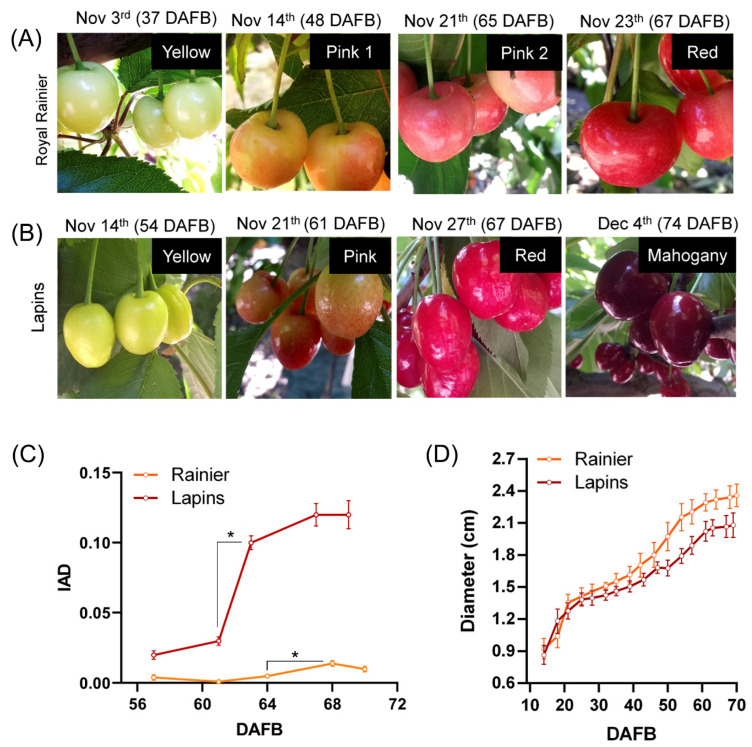
Variations in physiological parameters during sweet cherry fruit development in the bicolored cultivar ‘Royal Rainier’ and the dark-red cultivar ‘Lapins’. Color evolution stages in ‘Royal Rainier’ (**A**) and ‘Lapins’ (**B**). Pink 1, 2 and Red stages in ‘Royal Rainier’ and Pink, Red and Mahogany stages in ‘Lapins’ were utilized in the following analyses of this study. The Red stage in ‘Royal Rainier’ and the Mahogany stage in ‘Lapins’ corresponds to harvest time (14.68° Brix in ‘Lapins’ and 15.52° Brix in ‘Royal Rainier’). IAD (**C**) and equatorial diameter (**D**) evolution in ‘Royal Rainier’ and ‘Lapins’. In C and D, 20 fruits from four trees were measured (80 values per date). DAFB, Days After Full Bloom; IAD, Index of Absorbance Difference. Asterisks denote statistically significant differences (*p* < 0.0001) in the IAD parameter according to a two-tailed unpaired Student’s *t*-test.

This work used the dark-red ‘Lapins’ and the bicolored ‘Royal Rainier’ cultivars to dissect the molecular mechanism underlying color evolution differences. Both are midseason cultivars with a similar flowering date (mid-September in Chile). Both midseason cultivars present a double sigmoid growth curve (Figure 1). ‘Lapins’ color initiation occurred around 61 DAFB, whereas ‘Royal Rainier’ color change started at 48 DAFB. At the time of harvest (15.52° and 14.68° Brix in ‘Royal Rainier’ and ‘Lapins’, respectively), ‘Royal Rainier’ bicolored fruits developed a pink-red blush, and ‘Lapins’ acquired a homogenous mahogany color (Figure 1). ‘Royal Rainier’ fruits remained uncolored on the face that was shaded (Figure S1).

As shown in Figure 1C, the ‘Lapins’ cultivar showed higher IAD values than ‘Royal Rainier’ during all stages of fruit development. IAD values increase in ‘Lapins’ between 57 to 69 DAFB compared to the equivalent dates in ‘Royal Rainier’, where coloring occurs. On the other hand, we found that both cultivars showed a similar fruit size curve (Figure 1D), showing that differences in coloring are not linked to variations in the growth pattern. Thus, we further investigated if the accumulation of anthocyanins and other metabolites may differ between these sweet cherry cultivars. 

Analysis of anthocyanin levels showed that cyanidin-3-*O*-glucoside, peonidin-3-*O*-glucoside and peonidin-3-*O*-galactoside were not detected in the ‘Royal Rainier’ cultivar, but they increased during the different color stages of the ‘Lapins’ cultivar (Table 1). Notably, the total anthocyanin content was higher in ‘Lapins’ when compared with ‘Royal Rainier’ in all analyzed developmental stages. 

**Figure 2:**
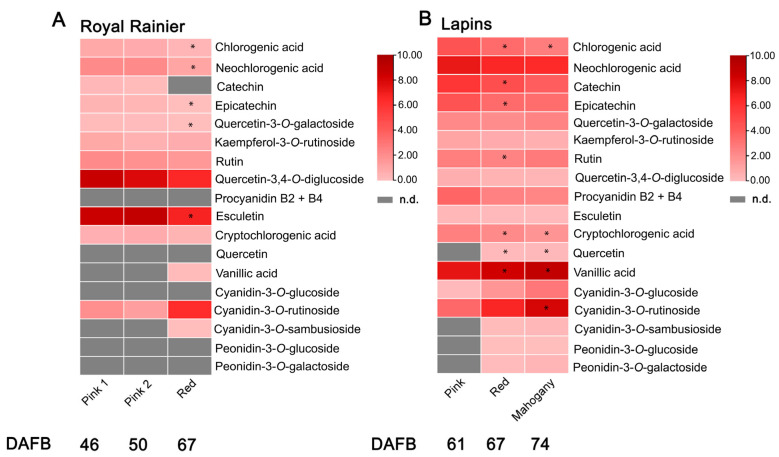
Accumulation dynamics of phenolic compounds in the bicolored ‘Royal Rainier’ and the dark-red cultivar ‘Lapins’. The heatmaps show the variation in phenolic compounds and anthocyanin levels determined in (**A**) ‘Royal Rainier’ and (**B**) ‘Lapins’ sweet cherry fruits during the color evolution of the indicated developmental stages. The corresponding DAFB (Days After Full Bloom) are shown below each stage. Heatmaps were generated using the log2 levels of each compound in the sweet cherry cultivars. The gray color indicates that the compound was not detected. The software TBTools (https://github.com/CJ-Chen/TBtools/releases, accessed on 21 June 2023) [27] was utilized for obtaining the graphs. Cultivar-specific stages of fruit coloration are defined in Figure 1. Asterisks denote statistically significant differences (*p* < 0.05) in the levels of each compound compared with the levels in the neighboring fruit coloration stage (e.g., Pink 1 vs. Pink 2 in ‘Royal Rainier’), using a two-tailed unpaired Student’s *t*-test.

Measurements of phenolic compounds and flavonoids showed that chlorogenic acid, neochlorogenic acid and catechins were detected in both cultivars. Remarkably, quercetin and procyanidin B2 + B4 were not detected in ‘Royal Rainier’ (Figure 2A), whereas both cultivars showed a decrease in several phenolics, including esculetin, chlorogenic acid, catechins and epicatechins during the color evolution associated with fruit development.

Vanillic acid was the most abundant phenolic compound in the ‘Lapins’ cultivar but was almost undetectable in ‘Royal Rainer’. Another relevant difference was the increased accumulation of quercetin-3,4-*O*-diglucoside and esculetin in ‘Royal Rainier’ compared to ‘Lapins’ (Figure 2) at all developmental stages analyzed.

**Figure 3:**
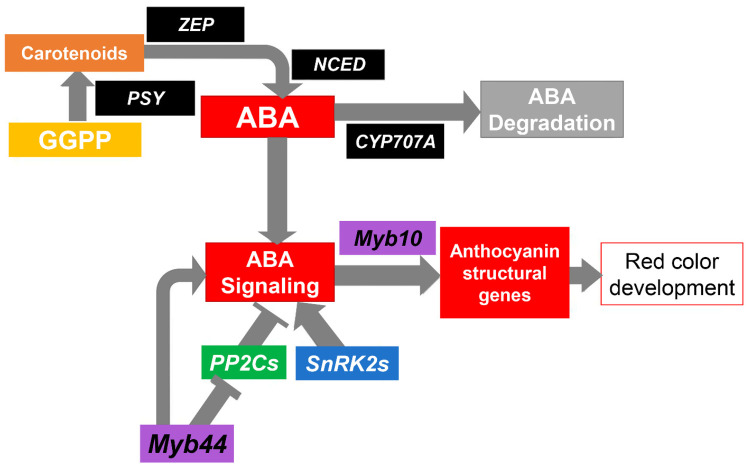
Schematic representation of the key steps and genes of ABA biosynthesis, catabolism, signaling and response, as well as their relationship with the red color formation.

In non-climacteric fruits, ABA plays a pivotal role in ripening, including color development. Several genes are involved in the synthesis and degradation of ABA; in turn, ABA modulates the expression of genes encoding MYB factors involved in controlling structural anthocyanin genes, such as *PavMYB10.1* (Figure 3).

**Figure 4:**
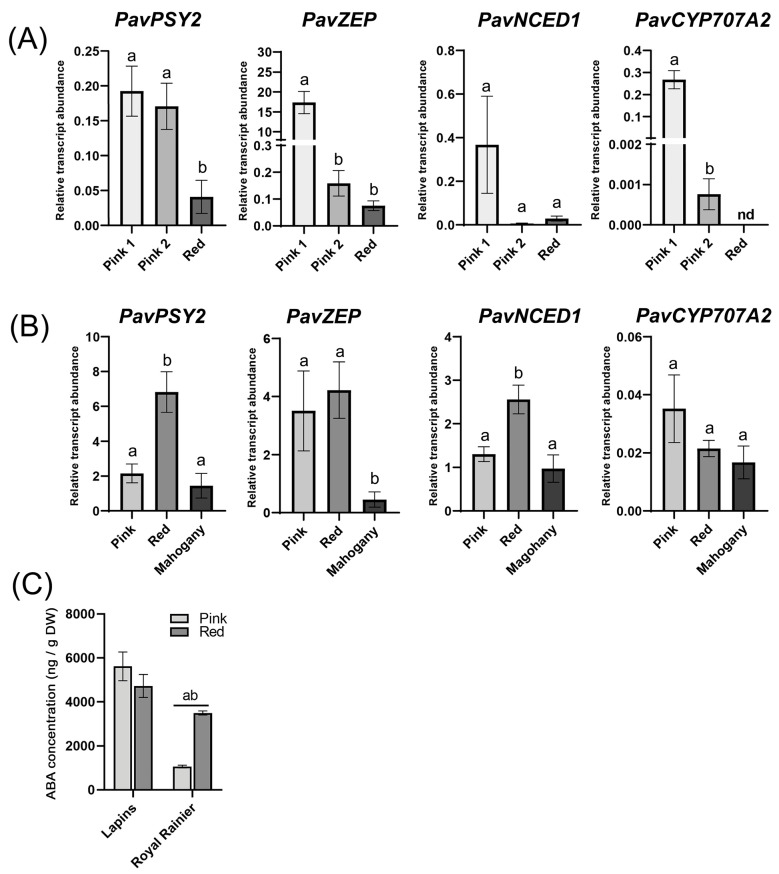
Transcript abundance of putative orthologues of ABA biosynthesis/degradation-related genes in ‘Royal Rainier’ (**A**) and ‘Lapins’ (**B**) sweet cherry fruits relative to *PavCAC* levels. Different letters are significant differences among all samples, according to a two-way ANOVA followed by a Tukey test, *p* < 0.05. (**C**) Endogenous dry weight (DW) ABA concentrations (expressed as ng/g DW) in ‘Lapins’ and ‘Royal Rainier’ sweet cherry fruits in two stages, pink (60 and 50 DAFB in ‘Lapins’ and ‘Royal Rainier’, respectively) and Red (67 DAFB in both cultivars). In (**A**–**C**), the average value and SEM from four trees of each developmental stage are depicted. For each stage, eight fruits per tree were pooled.

We found that the expressions of *PavPSY2*, *PavZEP*, and *PavNCED1* decreased in the transition from Pink 1 to Pink 2 in ‘Royal Rainier’ (Figure 4A) but increased in the ‘Lapins’ cultivar during the transition from Pink to Red (Figure 4B). However, the levels of *PavZEP* in the early stage of color initiation (Pink 1) were over five times higher in ‘Royal Rainier’ compared to ‘Lapins’ (Figure 4).

The transcript abundance of a gene involved in ABA degradation, *PavCYP707A2*, decreased in both cultivars in the transition from Pink to Red. However, *PavCYP707A2* transcript levels remained detectable in ‘Lapins’ fruits at the Mahogany and Red stages but were undetectable at the red stage in ‘Royal Rainier’ fruits. Finally, we also found that the ‘Lapins’ cultivar showed higher ABA levels when compared to ‘Royal Rainier’ in both the Pink and Red stages.

**Figure 5:**
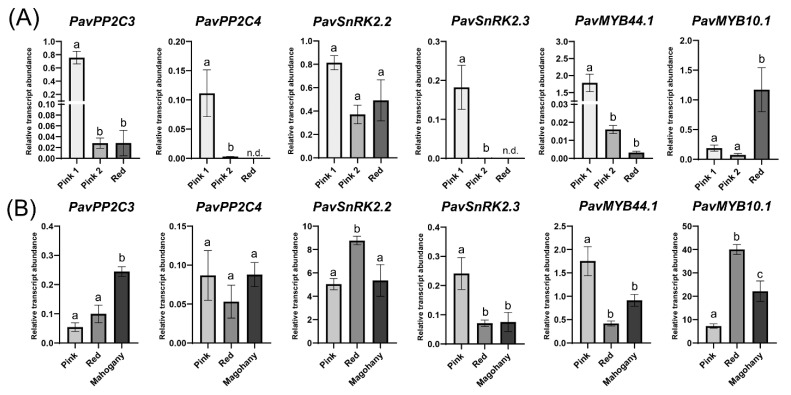
Transcript abundance of putative orthologues of ABA and response-related genes in ‘Royal Rainier’ (**A**) and ‘Lapins’ (**B**) sweet cherry fruits relative to *PavCAC* levels. Different letters are significant differences among all samples, according to a two-way ANOVA followed by a Tukey test, *p* < 0.05. The average value and SEM from four trees of each developmental stage are depicted. For each stage, eight fruits per tree were pooled.

Another point of control for anthocyanin accumulation is the expression of ABA signaling genes such as *PP2Cs* and *SnRKs* (Figure 3). Expression analysis of those genes showed a different expression trend in ‘Royal Rainier’ compared to ‘Lapins’ (Figure 5).

Notably, *PavPP2C3* and *PavSnRK2*.2 transcript abundance decreased in the transition from Pink 1 to Pink 2 in ‘Royal Rainier’, whereas in ‘Lapins’, *PavPP2C3* expression increased when transitioning from Red to Mahogany and *PavSnRK2*.2 increased from Pink to Red (Figure 5). 

MYB factors were also important in controlling the development of red coloration due to their role in regulating the expression of anthocyanin structural genes (Figure 3). In this regard, *PavMYB10.1* expression increased significantly in ‘Royal Rainier’ from the Pink 2 to Red stages and in the transition from Pink to Red stages in the ‘Lapins’ cultivar.

**Figure 6:**
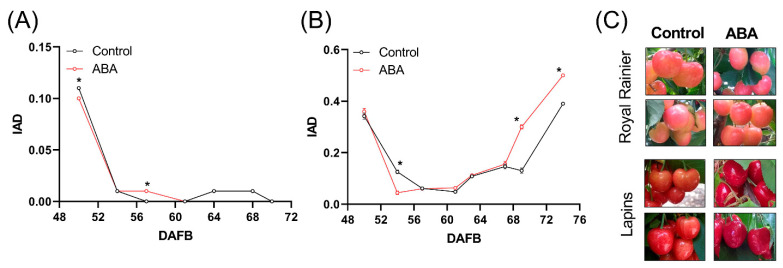
Effect of ABA treatment on fruit color and IAD parameter. Evolution of IAD parameter in ABA-treated fruits of ‘Royal Rainier’ (**A**) and ‘Lapins’ (**B**). In (**A**,**B**), 20 fruits from 4 trees were measured (80 values on each date per treatment). (**C**) Effect of ABA on the coloring of fruits. The pictures were taken 3 and 4 days before harvest in ‘Royal Rainier’ and ‘Lapins’, respectively. The ABA treatment was performed in the field at the onset of the color initiation (‘Royal Rainier’ 46 DAFB and ‘Lapins’ 56 DAFB) by hand spraying the trees with 400 ppm of the commercial product ProTone^®^ SL. Asterisks denote statistically significant differences (*p* < 0.05) in the IAD between control and ABA-treated groups at the given DAFB according to a two-tailed unpaired Student’s *t*-test.

We then evaluated if ABA treatment could differentially modulate the IAD parameter in ‘Lapins’ compared to ‘Royal Rainier’. In this regard, ABA did not increase the IAD parameter in ‘Royal Rainier’ (Figure 6A) as compared with ‘Lapins’, showing higher IAD values following ABA treatment (Figure 6B). Finally, we also observed that fruit coloration was increased in ‘Lapins’ but not in ‘Royal Rainier’ after ABA exposure (Figure 6C).

To better understand the temporality of the expression of these genes and the possibility that ABA could be directly regulating their expression (Figure 3 and Figure 4), the 5′ UTR regions of these genes were analyzed to identify potential ABA-responsive *cis*-elements using the *P. avium* Tieton genome as a reference. From all the evaluated genes, we found that all the 5′ UTR regions have ABA regulatory elements (ABREs) except for *PavMYB44.1*, *PavPP2C3* and *PavCYP707A1* (Figure S2).

**Figure 7:**
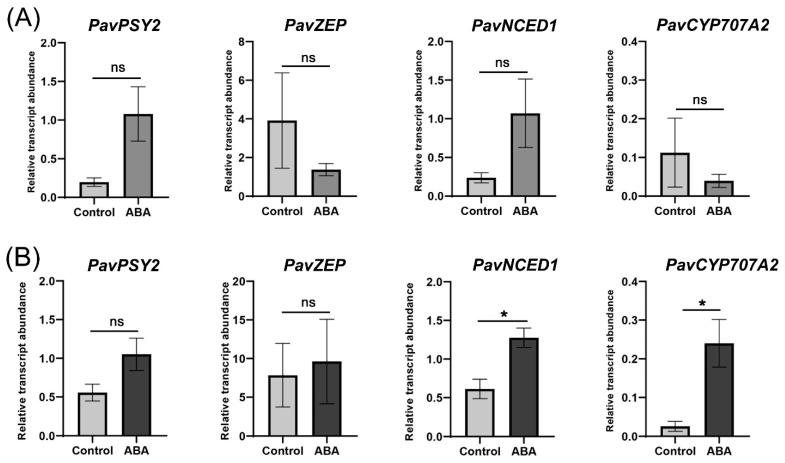
Effect of ABA treatment on transcript abundance of putative orthologs of ABA biosynthesis/degradation-related genes in ‘Royal Rainier’ (**A**) and ‘Lapins’ (**B**) sweet cherry fruits after two h of ABA treatment, relative to *PavCAC* levels. ABA treatment was performed as described in Figure 6. Asterisks represent significant differences between treatments, according to an unpaired Student *t*-test, *p* < 0.05. Average values and SEM from four trees of each developmental stage are depicted. For each stage, eight fruits per tree were pooled.

Expression of genes involved in ABA biosynthesis and catabolism, namely *PavNCED1* and *PavCYP707A2*, respectively, were significantly upregulated in ‘Lapins’ but not in the ‘Royal Rainier’ cultivar (Figure 7). Regarding other genes involved in the generation of ABA precursors such as *PavPSY2* or *PavZEP*, no significant differences were found in the ‘Royal Rainier’ nor the ‘Lapins’ cultivars upon ABA treatment.

**Figure 8:**
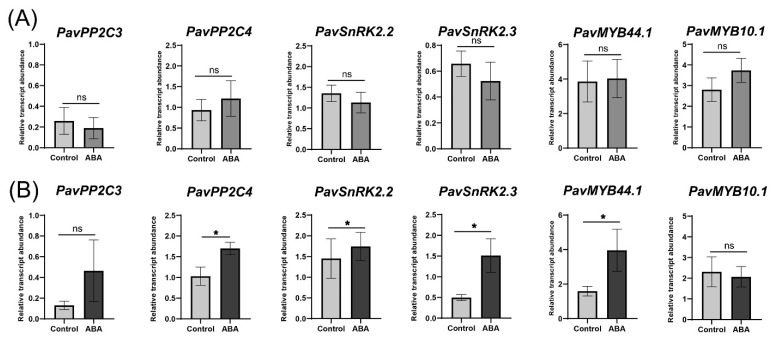
Effect of ABA treatment on the transcript abundance relative to *PavCAC* levels of putative orthologues of ABA signaling- and response-related genes in ‘Royal Rainier’ (**A**) and ‘Lapins’ (**B**) sweet cherry fruits after two h of ABA treatment. ABA treatment was performed as described in Figure 6. Asterisks represent significant differences between treatments, according to an unpaired Student *t*-test, *p* < 0.05. Average values and SEM from four trees of each developmental stage are depicted. For each stage, eight fruits per tree were pooled.

Investigating the responsiveness of different ABA signaling pathway-related genes, it was seen that *PavPP2C4* and *PavSnRK2.3* expression increased significantly following ABA treatment in ‘Lapins’ but not in the ‘Royal Rainier’ cultivar (Figure 8). Furthermore, there was an increase in *PavPP2C4*, *PavSnRK2.2* and *PavMYB44.1* transcript abundance after ABA treatment in the ‘Lapins’ cultivar, but not ‘Royal Rainier’ (Figure 8).

## 3. Methods

### 3.1. Plant Material

Sweet cherry (*Prunus avium* L.) adult trees of ‘Lapins’ and ‘Royal Rainier’ cultivars were selected from a commercial orchard in Rengo, Chile, Lon: O 70°43′6.78″ Lat: S 34°27′16.92″ during the 2017–2018 season. ‘Lapins’ trees were 15 years old, whereas ‘Royal Rainier’ trees were five years old. The trees were grown under similar conditions and agronomical practices. Trees were manually thinned to 4–5 fruits per cluster, resulting in similar fruit loads in all trees. Full flowering (50% open flowers in the trees) was set at zero time, corresponding to 0 DAFB (Days After Full Bloom). Four trees per cultivar were used for IAD (Index of Absorbance Difference) and diameter determination during fruit development and sampling for analysis, including transcript profiling, targeted metabolomics and hormone quantification. In the sampling, a pool of eight fruits was utilized.

### 3.2. ABA Treatment 

Eight trees per cultivar with similar growth status and vigor were randomly selected. Four trees were treated (hand sprayed to run-off) with 400 mg L^−1^ of S-ABA (ProTone^®^ SL, Valent, Libertyville, IL, USA), and four remained untreated. The trees were treated when fruits started to color (56 DAFB, 16 November 2017, in ‘Lapins’ and 46 DAFB, 12 November 2017 in ‘Royal Rainier’). The phenological stage for ABA treatment in both cultivars was 10% pink-blush straw-yellow fruits and 90% straw-yellow fruits in the tree. Color Absorbance Index (IAD) and diameter were measured in fruits on ABA-treated and control trees, collected and frozen in liquid nitrogen, then stored at −80 °C for further analyses.

### 3.3. Physiological Evaluations

Four trees per cultivar were randomly selected in the orchard. For the IAD and equatorial diameter curves, 20 fruits per tree were measured every 2–5 days. The equatorial diameter from the suture was measured with a caliper, and IAD was measured using VIS/NIF device Cherry Meter (T.R.^®^ Turoni, Italy). IAD and equatorial diameter curves were generated using the Software GraphPad Prism v6.0.

### 3.4. Targeted Metabolomics of Phenolic Compounds 

Targeted metabolic analyses of phenolic compounds were performed as reported previously [19,21,28]. Around 150 mg of each lyophilized sweet cherry fruit sample was transferred into 15 mL falcon tubes, and a volume of 4 mL of 80% methanol was then added. The samples were sonicated for 20 min and mixed by orbital shaking for three hours at ambient temperature. The extraction was kept overnight at 4 °C in the dark. After that, samples were centrifuged for 10 min (1800× *g*; 4 °C) and filtered using 0.22 μm PTFE (Polytetrafluoroethylene) membranes. Samples were stored at −80 °C until analysis. The tissue extractions were conducted in triplicate for each developmental stage. Targeted Ultra High-Performance Liquid Chromatography (UHPLC) was performed on a Waters Acquity System (Milford, MA, USA) having a binary pump, an online vacuum degasser, an autosampler and a column compartment. The compounds were separated on a Waters Acquity HSS T3 column 1.8 μm, 100 mm × 2.1 mm, at 40 °C. The analysis of phenolic compounds was performed according to Vhrovsek et al. (2012) [29]. The analysis of anthocyanins was performed as described by Arapitsas et al. (2012) [30]. For Mass Spectrometry (MS) detection, a Waters Xevo TQMS instrument equipped with an ElectroSpray (ESI) source was used. Data processing was performed using the Mass Lynx Target Lynx Application Manager (Waters, Milford, MA, USA). The log2 value of compound concentration expressed as mg/100 g FW was used to generate the heatmaps using the TBTools software [31]. Statistical differences between neighboring cultivar-specific fruit coloration stages (Figure 1) were determined using a two-tailed unpaired Student’s *t*-test.

### 3.5. Hormone Quantification

For ABA measurements, 50 mg of lyophilized fruit pericarp was ground and resuspended in 80% methanol—1% acetic acid solution containing internal standards (deuterium-labeled hormones; OlChemim Ltd., Olomouc, Czech Republic). The mix was shaken for one hour at 4 °C, and the extracted fraction was incubated overnight at −20 °C. The tissue extractions were conducted in triplicate for each developmental stage. Samples were centrifuged, and the supernatant was vacuum dried and dissolved in 1% acetic acid. A reverse-phase column (OasisHLB) was used, and the eluate was dried and dissolved in a solution of 5% acetonitrile and 1% acetic acid. An autosampler and reverse-phase UHPLC chromatography column, 2.6 μm Accucore RP-MS, 100 mm × 2.1 mm (Thermo Fisher Scientific, San Diego, CA, USA) was used. ABA was separated through a gradient of acetonitrile (2–55%) containing 0.05% acetic acid at a rate of 400 μL/min over 22 min and detected in a Q-Exactive mass spectrometer (Orbitrap detector; ThermoFisher Scientific; San Diego, CA, USA). Targeted Selected Ion Monitoring and Electrospray Ionization in the negative mode were used to detect ABA. Measurements were performed using external calibration curves with the Xcalibur 4.0 and TraceFinder 4.1 SP1.

### 3.6. RNA Extraction, cDNA Synthesis and RT-qPCR Analyses

Total RNA was isolated from 1 g of liquid nitrogen ground fruit tissues (mesocarp and exocarp enriched tissue) using the CTAB method with minor modifications, according to Meisel et al. (2005) [32]. Genomic DNA traces were eliminated using DNAse TURBO™ (Thermo Fisher Scientific, CA, USA). Purity values (A260/230 and A260/A280) ranged between 1.8–2.2 in all the samples. For cDNA synthesis, 1 μg of total RNA was retrotranscribed using the First Strand cDNA Synthesis System Kit (Thermo Fisher Scientific, Carlsbad, CA, USA). Relative quantifications of sweet cherry transcripts were determined RT-qPCR using two technical replicates and four biological replicates. Primers were selected from the literature [10,33,34,35]. In the case of *PavPSY2* and *PavZEP*, the putative orthologues in sweet cherry were identified by bidirectional BLAST between the Arabidopsis proteins (obtained from ThaleMine https://bar.utoronto.ca/thalemine/begin.do, accessed on 1 October 2017) and the sweet cherry genome cv. Satonishiki [36]. The primers were designed by using the NCBI tool. A Basic Local Alignment Search Tool (BLAST) was performed against the sweet cherry genome (NCBI databases) to confirm that these primers align only with the genes of interest. The efficiency of these primers was determined by the LinRegPCR program [37]. Primer efficiency was considered for relative transcript abundance calculations, as indicated by Pfaffl (2001) [38]. The list of the primers is detailed in Table S1. The RT-qPCR reaction was performed on a QIAGEN Rotor-Gene Q, using the Rotor-Gene Q Series software version 2.1.0. The reactions were performed using the Takyon™ ROX SYBR 2X MasterMix dTTP blue—Master Mix (Eurogentec, Seraing, Belgium). RT-qPCR analyses were performed using the recommended conditions and “Golden Rules of Quantitative PCR” [39]; *PavCAC*, *PavTEF2* and *PavACT1* genes were used as normalizers according to Alkio et al. (2014) [33]. For the relative abundance calculations, *PavCAC* was used since it had the most negligible variation between samples (<1.5 Cq). All the graphs were made with the GraphPad software Prism version 6.0e. For RT-qPCR analyses, the statistical differences between different developmental stages or between control and ABA treatments were determined by two-way Analysis of Variance (ANOVA) followed by a Tukey test using the software GraphPad Prism 6.0.

### 3.7. In Silico Analyses of UTRs

A region of 2500 bp upstream of transcription start was analyzed for each gene using the web tool PlantCare (https://bioinformatics.psb.ugent.be/webtools/plantcare/html/, accessed on 21 June 2023) [40]. Data obtained from this website were then imported to the software TBTools [31], which was used to generate the position of the different regulatory elements along the 5 UTR of each gene.

## 4. Discussion

### 4.1. Differences between Dark-Red and Bicolored Fruits at the Metabolic and Gene Expression Level 

A dark-red sweet cherry cultivar was compared to a bicolored cultivar to further understand the fruit coloring process during ripening. Total anthocyanins content in fruits was lower in the bicolored cultivar than in the dark-red cultivar (86.25 mg/100 g FW in ‘Royal Rainier’ and 530.47 mg/100 g FW in ‘Lapins’; see Table 1). These results are consistent with similar findings comparing ‘Lapins’ and ‘Rainier’ cultivars (‘Rainier’ is a bicolored cultivar, similar to ‘Royal Rainier’) [10]. Jin et al. (2016) and Liu et al. (2013) [9,10] found that the expression of most anthocyanin structural genes was higher in the dark-red cultivar than in the bicolored cultivar, including *PavUFGT* and *PavDFR*.

Directed metabolic profiling using UHPLC-MS/MS revealed that some minor anthocyanins present in ‘Lapins’ were not detected in ‘Royal Rainier’, as in the case of cyanidin-3-*O*-glucoside, peonidin-3-*O*-glucoside and peonidin-3-*O*-galactoside. Cyanidin-3-*O*-rutinoside was the most abundant anthocyanin in both cultivars (Table 1, Figure 2). Jia et al. (2019) [41], comparing 12 sweet cherry cultivars, found that cyanidin-3-*O*-rutinoside was the most abundant anthocyanin in all the genotypes, including dark and bicolored ones. Color differences between ‘Royal Rainier’ and ‘Lapins’ might be related to the anthocyanins absent in ‘Royal Rainier’ plus the lower levels of cyanidin-3-*O*-rutinoside. For instance, red stripes in apple fruit peel have been reported to have a higher anthocyanin accumulation than green stripes, which is associated with higher cyanidin-3-*O*-galactoside content [14]. Regarding the dynamics of anthocyanins accumulation, the anthocyanins levels of ‘Royal Rainier’ and ‘Lapins’ increased at the final stage of ripening (Figure 2). In terms of phenolics and other flavonoids, several compounds, including flavan-3-ols, flavonols (e.g., rutin), vanillic acid, and neochlorogenic acid, among others, were detected in both cultivars. These compounds are generally characteristic of sweet cherries [41]. Quercetin and procyanidin B2 + B4 were not detected in ‘Royal Rainier’ (Figure 2A). Furthermore, both cultivars shared the decrease pattern of several phenolics, such as esculetin, chlorogenic acid, catechins and epicatechins during fruit development, similar to what we have reported previously [22]. Interestingly, the flavonol glycoside rutin decreased during the coloring process in ‘Royal Rainier’ but accumulated in the dark-red cultivar ‘Lapins’ (Figure 2).

Here, we found that less anthocyanin content in bicolored ‘Royal Rainier’ was accompanied by lower transcript abundance of *PavMYB10.1* (Figure 4). This is similar to a previous report in sweet cherry, where ‘Rainier’ fruits had less total anthocyanin content and less expression of *PavMYB10.1* than ‘Lapins’ fruits [10]. *PavMYB10* transcript levels were also lower in ‘Rainier’ than in ‘Stella’ dark-red fruits [24]. 

MYB genes are crucial in the control of anthocyanin structural genes. In grapes, a retrotransposon-induced mutation in *VvmybA1* was associated with the absence of anthocyanins in fruits of white cultivars [42]. In strawberry, *FaMYB10* RNAi fruits showed less coloration and anthocyanin content [20]. Downregulation of *PacMYBA* resulted in less colored sweet cherries [18]. In addition, a homozygous deletion in the third exon of *PavMYB10* in sweet cherry has been associated with the bicolored fruit phenotype [10]. 

Different alleles of *PavMYB10.1* are associated with different skin colors at the mature stage in sweet cherry [10]. The *PavMYB10.1b* allele has a 1-bp deletion in the third exon, whereas *PavMYB10.1a* has no deletion. Jin et al. (2016) [10] found that the dark-red cultivar ‘Lapins’ was heterozygous (*PavMYB10.1a* and *PavMYB10.1b*), whereas the bicolored cultivar ‘Rainier’ was homozygous for *PavMYB10.1b*. However, a functional demonstration that *PavMYB10.1* is the unique or most important genetic factor controlling coloring has not been performed, for instance, by overexpressing the non-deleted allele of this gene in bicolored sweet cherry fruits. Here, we argue that other genetic factors participate in color evolution and focus on the ABA pathway to explain, at least in part, differences between dark-red and bicolored cherries. 

Our results suggest that the ABA pathway is upstream of the action of the MYB genes. In line with this, some reports show that treatment with ABA synthesis inhibitors affects the expression of MYB genes [18,20]. In strawberry, silencing of a 9-*cis*-epoxycarotenoid dioxygenase gene, *FaNCED1*, or the putative ABA receptor *FaCHLH/ABAR* in fruits, caused *FaMYB10* downregulation [20]. This result suggests that the expression of this MYB gene, crucial for anthocyanin synthesis in strawberry, depends on ABA production and perception. Similarly, in sweet cherry, silencing of *PacNCED1* inhibits the accumulation of anthocyanins and reduces *PacMYBA* (*PavMYB10.1*) expression [18]; therefore, this gene can be considered an ABA-response gene. Given the relevance of ABA in controlling genes involved in the color evolution process, we wondered if the expression of ABA homeostasis- and signaling-related genes depicted in Figure 3 was different between dark-red and bicolored fruits.

In general, ABA biosynthesis-related genes had higher expression in ‘Lapins’ than in ‘Royal Rainier’ in all stages analyzed (Figure 4A,B), correlating with higher endogenous ABA content in the Pink and Red stages (Figure 4C). *PavPSY*, *PavZEP* and *PavNCED1* decreased dramatically from the Pink to the Red stage in the bicolored ‘Royal Rainier’. In contrast, these genes increased in the dark-red ‘Lapins’ (Figure 4A,B). Regarding signaling genes, at the Pink stage (Pink in ‘Lapins’ and Pink 1 in ‘Royal Rainier’), the expression levels were similar in the fruits of both cultivars, except for *PavSnRK2.2* (Figure 5), and expression differences were displayed after this stage. These findings suggest that the Pink stage is an inflection point for color development, and differences at the molecular level between cultivars become apparent after this point. In fact, after the Pink stage, a substantial reduction in gene expression of signaling genes occurs in ‘Royal Rainier’ but not in ‘Lapins’, possibly suggesting that in addition to having less ABA content, pink bicolor cultivars could have a more attenuated ABA pathway.

### 4.2. Dark-Red and Bicolored Fruits Respond Differently to ABA 

In sweet cherry and strawberry, transient silencing of a 9-*cis*-epoxycarotenoid dioxygenase gene resulted in uncolored fruits with less anthocyanin content [18,43], showing that ABA is essential in the control of color in non-climacteric species. Jia et al. (2011) [43] found that the uncolored phenotype of the *FaNCED1* RNAi fruits, but not the *FaCHLH/ABAR* RNAi fruits, could be rescued by exogenous ABA. These findings suggest that perception or signaling is probably defective when ABA cannot reverse an uncolored phenotype. In line with this model, we found that fruit color and IAD parameters did not change in response to ABA treatment in ‘Royal Rainier’. It was previously reported that ‘Bing’ and ‘Lapins’ dark-red cultivars, but not ‘Royal Rainier’, increased this anthocyanin content-related parameter upon ABA treatment of the tree canopy [44]. It seems that ABA unresponsiveness is characteristic of bicolored sweet cherry cultivars but does not necessarily occur in other pink fruits. For instance, in the wild Chilean strawberry *Fragaria chiloensis,* which develops a white/pink receptacle, ABA enhances color and anthocyanin accumulation [28].

Gene expression associated with ABA biosynthesis in response to ABA treatment is similar in ‘Royal Rainier’ and ‘Lapins’. However, ABA treatment increases the expression of *PavCYP707A2*, involved in ABA degradation, only in the dark-red cultivar ‘Lapins’ (Figure 7). This was not expected as the −2500 bp region upstream of the transcription start site does not contain ABREs (Figure S2). The ABA effect on this gene is possibly indirect and unrelated to its promotor’s modulation. As shown in Figure 8, ABA treatment increased the expression of signaling genes *PavPP2C3*, *PavPP2C4*, *PavSnRK2.2* and *PavSnRK2.3* only in ‘Lapins’ similar to those reported by Kuhn et al. (2021) [13], except for *PavSnRK2.3*. Our results suggest that ‘Royal Rainier’ is insensitive to ABA treatment, possibly for having a more dampened ABA pathway at the Red stage.

Other factors, different from MYB and ABA pathway genes, are involved in fruit color. Light is essential for anthocyanin production mediated by MYB genes [27]. Guo et al. (2018) [26] performed a bagging treatment that excluded light in sweet cherry fruits and observed the effect on fruit color of the bicolored cultivar ‘Rainier’ and the dark-colored cultivar ‘Hongdeng’. The light was key for color development in ‘Rainier’, showing significantly higher levels of anthocyanins in the unbagged vs. bagged fruits or with bag removal.

In contrast, the bagging treatment did not affect color in ‘Hongdeng’; therefore, it was concluded that this cultivar was only slightly light dependent [26]. We differ on this interpretation since transcriptome analysis of ‘Hongdeng’ fruits exposed to the light vs. bagged showed hundreds of DEGs, thus supporting that this cultivar is light responsive. Thus, we argue that dark-red ‘Hongdeng’ is not necessarily insensitive to light; otherwise, light responsiveness could be masked by a strong production of anthocyanins controlled by ABA and MYB genes. Under this rationale, it would be interesting to generate *PavNCED1* or *PavMYB10.1* RNAi fruits of a dark-red cultivar and then assess the contribution of the light in color evolution. 

Our results show that the ABA pathway is affected in ‘Royal Rainier’; therefore, light is the primary signal controlling color, explaining why the shaded face does not develop colored further (Figure S2). A crosstalk between light and ABA pathway could enhance a dampened ABA pathway in ‘Rainier’. In this line, light and ABA increased the expression of *FaMYB10* in red strawberry fruits [20]. Identifying genetic points of convergence of light and ABA pathway genes would be interesting.

**Figure 9:**
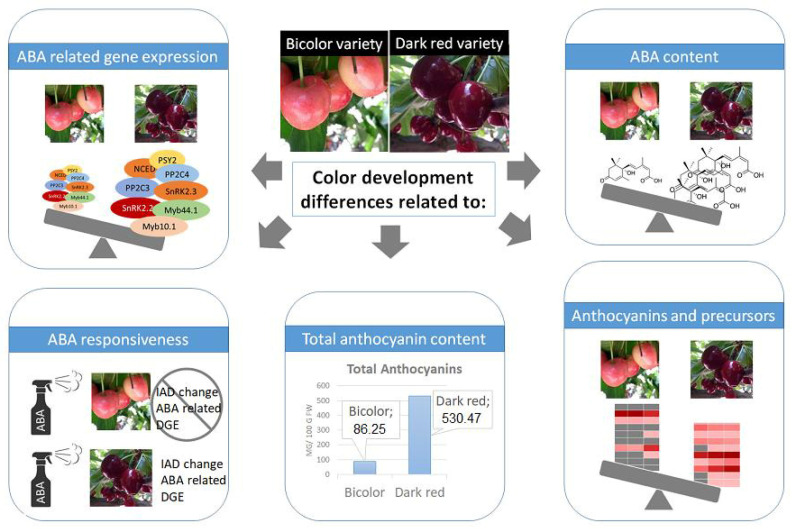
Schematic representation of the main finding of this work regarding differences in ABA gene expression; ABA, anthocyanin and other phenolic precursors content; and differential response to ABA in field trials between dark-colored and bicolored varieties.

A schematic representation of the main finding of this work, in terms of differential ABA gene expression; ABA, anthocyanin, and other phenolic precursors content; and differential response to ABA in field trials is shown in Figure 9. Future perspectives also include analysis of the promoters of ABA pathway genes and MYB genes comparing cultivars with different colors. Finally, the role of methylation could also be explored. In apple cultivars, red stripes in the fruit peel accumulated more anthocyanins and had more expression of *MYB10* than green stripes [14]. These authors found that cytosine methylation in the promoter of *MYB10* was enriched in green stripes. Methylation could also be modulating the expression of ABA pathway genes. Transcriptomic and methylome analysis of uncolored face vs. colored face of bicolored sweet cherry fruits in a combination of bagging and ABA treatments could help to determine more accurately the contribution of light and ABA to coloring.

Regarding the novelty and contribution of this work, this is the first attempt to unravel metabolomic and ABA-related gene expression differences between bicolored and dark-red cultivars. Additionally, the effect of ABA in both cultivars is contrasted at the gene expression level for the first time.

## Figures and Tables

**Table 1:** Anthocyanin content during fruit color evolution of ‘Royal Rainier’ (stages Pink 1, Pink 2 and Red) compared to ‘Lapins’ (Pink, Red and Mahogany). Methanol-extracted anthocyanins were quantified using Targeted Ultra High-Performance Liquid Chromatography (UHPLC). The values are expressed in the table as mean ± SEM of mg/100 g of fresh weight tissue (FW). Not detected compounds are expressed as n.d. Cultivar-specific stages of fruit coloration are defined in Figure 1. A two-tailed unpaired Student’s *t*-test was performed to determine the statistical difference between neighboring fruit coloration stages (e.g., Pink 1 vs. Pink 2 in ‘Royal Rainier’). The letters a, b and c represent statistical differences between neighboring stages.

	*‘Royal Rainier’* (mg/100 g FW)			*‘Lapins’* (mg/100 g FW)		
	Pink 1	Pink 2	Red	Pink	Red	Mahogany
*Cyanidin-3-O-glucoside*	n.d.	n.d.	n.d.	0.08 ± 0.02 ^a^	2.44 ± 0.48 ^b^	8.51 ± 0.39 ^c^
*Cyanidin-3-O-rutinoside*	2.89 ± 0.72 ^a^	1.47 ± 0.45 ^a^	86.2 ± 45.22 ^a^	13.98 ± 2.5 ^a^	178.56 ± 32.04 ^b^	521.56 ± 20.33 ^c^
*Cyanidin-3-O-Sambubioside*	n.d.	n.d.	0.05 ± 0.04	n.d.	0.04 ± 0.01 ^a^	0.17 ± 0.01 ^b^
*Peonidin-3-O-glucoside*	n.d.	n.d.	n.d.	n.d.	<0.01 ^a^	<0.01 ^b^
*Peonidin-3-O-galactoside*	n.d.	n.d.	n.d.	n.d.	0.02 ± 0.01 ^a^	0.23 ± 0.12 ^a^
*Total Anthocyanins*	2.89 ± 0.72 ^a^	1.47 ± 0.45 ^a^	86.25 ± 45.26 ^a^	14.06 ± 2.6 ^a^	181.24 ± 32.54 ^b^	530.47076 ± 20.85 ^c^

## Data Availability

Data sharing does not apply to this article as all newly obtained data are already contained within this article and the Supplementary Material.

## Supplementary Materials

The following supporting information can be downloaded at: https://www.mdpi.com/article/10.3390/plants12132493/s1, Figure S1: Representative image of biocolored Royal Rainer fruits during ripening; Figure S2: ABA responsive elements (ABREs) found in the ~2500 bp region of the indicated genes; Table S1: List of primers used for this study.

## Abbreviations

ABA: abscisic acid; IAD: index of absorbance difference; DAFB: days after full bloom; UHPLC-MS/MS: ultra high-performance liquid chromatography-tandem mass spectrometry.
