# Supplementary material for: ABA Biosynthesis- and Signaling-Related Gene Expression Differences between Sweet Cherry Fruits Suggest Attenuation of ABA Pathway in Bicolored Cultivars
Source: Plants (Basel). 2023 Jun 29;12(13):2493. doi: 10.3390/plants12132493 (PMC10346923; doi:10.3390/plants12132493)
Supplement: Supplementary file 1 [file plants-12-02493-s001.zip › plants-2434183-supplementary.pdf]

Supplementary Tables

Table S1. List of primers used for this study

Gene symbol	Name	Sequence	Tm ºC	Accession Number	Reference
PavMYB10.1	PavMYB10.1-F	5’ - ACGCCATCACAAACATCACC - 3’	59.07	KP455683.1	Li et al. 2015
	PavMYB10.1-R	5’ - GTCCTTCTGAACATTGGTACACT - 3’	60.05		
PavMYB44.1	PavMYB44.1-F	5’ - GTGAGATGGGGTACGATCAGC - 3’	59.07	XM_008222055.1	Li et al. 2015
	PavMYB44.1-R	5’ - GGCGTTTCTAATGGCTTCGG- 3’	60.05		
PavPSY2	PavPSY2-F	5’ – GCCTACCATATTAGACGAAGCCA - 3’	62.9	XM_021969171.1	This work
	PavPSY2-R	5’ –GGGTGAGCAAAGAGCAATGTC- 3’	61.3		
PavZEP	PavZEP-F	5’ –CCGATATTGAGGGGAAGCGA- 3’	60.5	XM_021969154.1	This work
	PavZEP-R	5’ –TTCCTTCTCAACACTCCCTGG- 3’	61.3		
PavNCED1	PavNCED1-F	5’ - CTCCAGAGTTCCGTATGGTTTTC - 3’	59.07	GQ913652.1	Li et al. 2015
	PavNCED1-R	5’ - TAGCTTCCCACAGGTAATTGTCC - 3’	60.05		
PavCYP707A2	PavCYP707A2-F	5’ - GAACAATCACCACCACAAAGAACTG - 3’	60.96	GU559988.1	Li et al. 2015
	PavCYP707A2-R	5’ - CTTGCCGAGACCGATTTATTGTATG - 3’	60.33		
PP2C3	PavPP2C3-F	5’ - TGGAGTTTTCCGGCTCTTCC - 3’	59.96	XM_021977988.1	Li et al. 2021
	PavPP2C3-R	5’ - TCGGCAAAACGATGGGTGTA - 3’	59.96		
PP2C4	PavPP2C4-F	5’ - GGAGTCTAAATCTGTGCGTGTGGAC - 3’	63.55	AF411074.1	Wang et al. 2015
	PavPP2C4-R	5’ - AAACTCCATAGAAGTGGGCTCCATT - 3’	62.06		
SnRK2.2	PavSnRK2.2-F	5’ - AAACTATGCGGCAGTGGAGCG - 3’	63.6	KY780374.1	Wang et al. 2015
	PavSnRK2.2-R	5’ - ACATCGACTTCGGAGTCCAAATCAG - 3’	62.85		
SnRK2.3	PavSnRK2.3-F	5’ - GTTGCTACAGCAATGGAGCG - 3’	59.6	KY780377.1	Kuhn et al. 2021
	PavSnRK2.3-R	5’ - TGGGATGCTTCAAGGACCTG - 3’	59.67		
CAC	PavCAC-F	5’ - GCGTGCCAGACTGACCTT - 3’	59.97	XM_021952533.1	Alkio et al. 2012
	PavCAC-R	5’ - GGCGAGCGTGACATATCTAACC - 3’	61.17		

Supplementary Figures

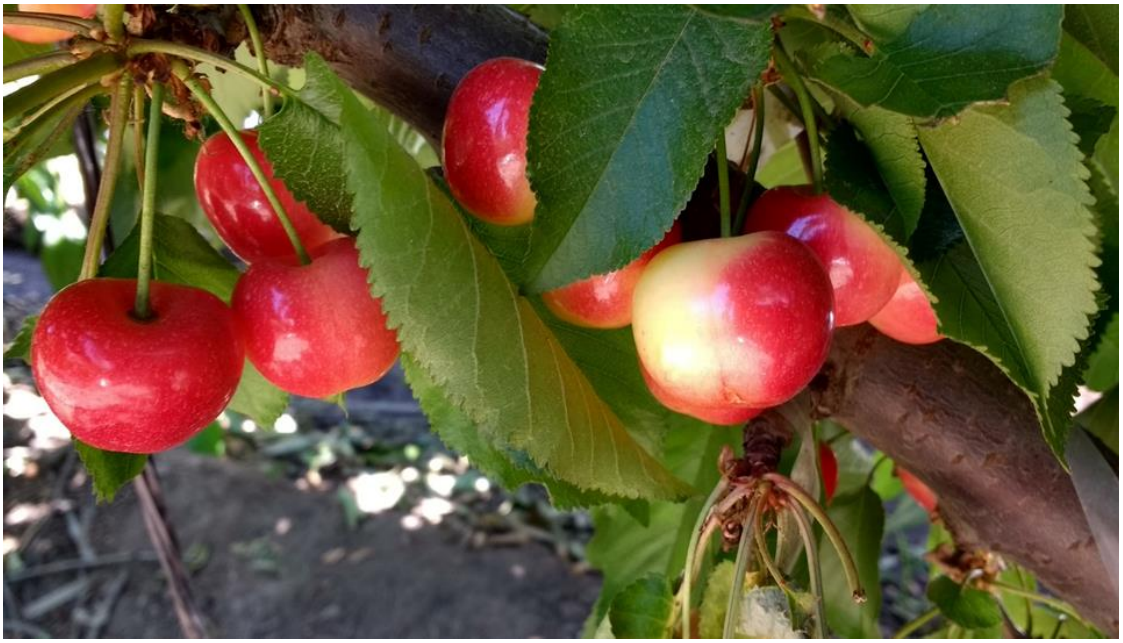

Figure S1. Representative image of bicolored Royal Rainier fruits during ripening.

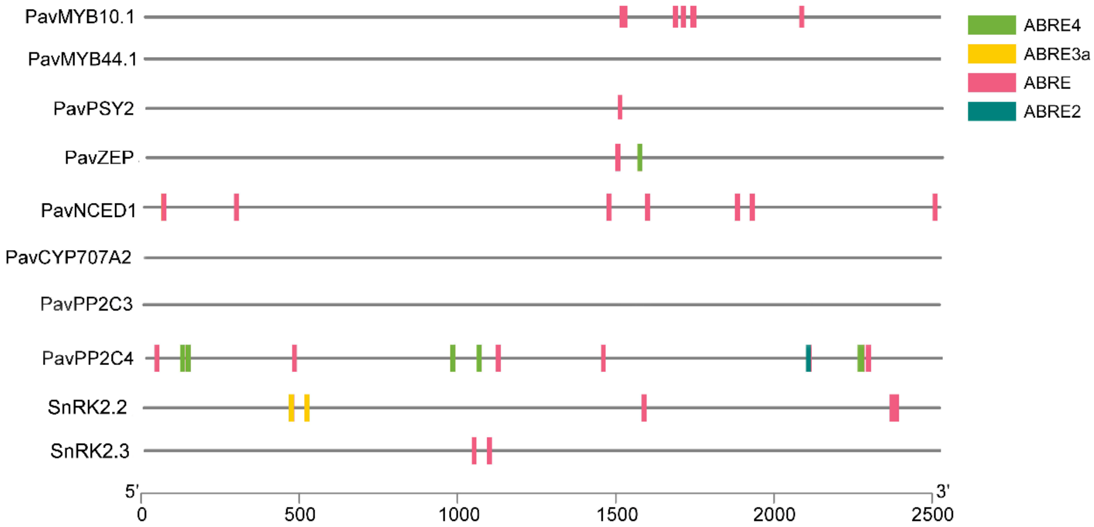

Figure S2. ABA responsive elements (ABREs) found in the -2500 bp region of the indicated genes. Sequences of genes from *Prunus avium* genome were obtained in the *Prunus avium* Tieton genome v2.0 transcripts database then used as query in JBrowse to identify the corresponding sequence. The sequences were analyzed in PlantCare. Illustration of the position of each different regulatory element was generated with the software TBTools.
